# Assessing and enhancing pediatric residents’ knowledge and skills in tracheostomy care through simulation-based training

**DOI:** 10.3389/fped.2025.1551517

**Published:** 2025-07-15

**Authors:** Hasan Ghandourah, Nada Townsi, Meral Abualjdyel, Muhammed A. Khan, Narvanie Seebran, Salma Mowalled, Winifred Mary El Jamal, Haifaa Kashqari, Saif Selati

**Affiliations:** ^1^ Department of Paediatrics, King Abdulaziz Medical City, National Guard Health Affairs, Jeddah, Saudi Arabia; ^2^ College of Medicine, King Saud Bin Abdulaziz University for Health Sciences, Jeddah, Saudi Arabia; ^3^ King Abdullah International Medical Research Center, Jeddah, Saudi Arabia; ^4^ Nursing Education Department, King Abdulaziz Medical City, National Guard Health Affairs, Jeddah, Saudi Arabia

**Keywords:** confidence, knowledge, medical education, pediatric residents, simulation, skills, tracheostomy

## Abstract

**Background and Objectives:**

The pediatric residency program in Saudi Arabia currently does not include tracheostomy care as a competency. Research has indicated that nonsurgical residents have limited knowledge of tracheostomy management. This study aimed to establish the need for pediatric residents to be trained in tracheostomy care and the effectiveness of having such a training program.

**Methods:**

This cross-sectional and interventional study included all pediatric residents who completed a self-assessment questionnaire of confidence levels, an objective knowledge assessment, and a hands-on routine pediatric tracheostomy tube change assessment on a mannequin. A targeted tracheostomy workshop was tailored to pediatric residents using didactic presentations and hands-on simulation practice.

**Results:**

Forty-two residents participated in this study. The residents had limited experience with tracheostomy care, as they had not received formal training in this area nor practiced changing a tracheostomy tube independently. Thirty-one residents (73.81%) felt incompetent in assessing patients with a tracheostomy, and 39 (92.86%) lacked confidence in performing tracheostomy tube change care. The levels of confidence among the residents, knowledge regarding management of tracheostomy, and clinical expertise in tracheostomy tube change was significantly higher after the workshop than their scores measured prior to the workshop.

**Conclusion:**

This study illustrates that the healthcare professionals in pediatrics are not self-assured and knowledgeable in the field of tracheostomy care and, thereby, establishes the necessity of a specific tracheostomy educational program to enhance the self-assurance, knowledge, and competency in performing the practice.

## 1 Introduction

Tracheostomy is among the most ancient surgical procedures and is the most common procedure for critically ill patients. In the United States, the number of children undergoing tracheostomy is steadily increasing due to advances in medical technology, resulting in longer hospital stays (1). Zhang et al. (2) conducted a detailed economic analysis of pediatric tracheostomy care, demonstrating that the total amount of care used over five years was $321 million, with inpatient hospitalizations accounting for 96% of these costs. In recent decades, advances in complex care medicine have significantly changed the indications and characteristics of children requiring a tracheostomy (3, 4).

Today, tracheostomy care is particularly common among premature infants and children who rely on ventilators, which require tracheostomy and related medical technologies, for the expiration of their long-term life (5, 6). Many of these incidents originate from correctable deficiencies in the knowledge and skills of family members and healthcare personnel. The timely intervention and effective management of these critical situations are crucial to mitigate risks (7, 8).

Safe and effective care for children with tracheostomies requires high confidence, knowledge, and skills. Unfortunately, nonsurgical healthcare providers often receive inadequate training in this area. According to a recent cross-sectional study, most nurses in Saudi Arabia are not well educated (9). Furthermore, the pediatric curriculum established by the Saudi Commission for Health Specialties does not recognize routine or emergency tracheostomy tube changes as essential competencies (10). Consequently, our pediatric residency program fails to adequately prepare residents to handle these critical situations. Residents are often the first responders but they lack the confidence and knowledge required for effective tracheostomy management (11–14).

New studies, including the study of Mehta et al. (15), have demonstrated the effectiveness of targeted educational workshops and simulated human classes that substantially increase the knowledge and skills of pediatric residents in tracheostomy care. This underlines the reason for a simulation program targeted at the pediatric segment.

No published study to date has reviewed the knowledge or confidence of Saudi pediatric residents in the care of tracheostomy tubes, despite the important role played by residents as the first responders to hospital emergencies involving tracheostomy. To address this gap, we assessed the knowledge, confidence, and skills of pediatric residents accomplished in a large tertiary care hospital. Furthermore, we discussed the impact of an integrated simulation-based training workshop on increasing their competence and confidence in tracheostomy management.

## 2 Materials and methods

The King Abdullah International Medical Research Center approved this cross-sectional, interventional study (reference number IRBC/RJ20/206/J), which was conducted at King Abdulaziz Medical City, National Guard Health Affairs, Jeddah, Saudi Arabia. The Saudi Commission for Health Specialties has accredited over 25 training residency programs in King Abdulaziz Medical City. The large pediatric services of the hospital, including the pediatric otolaryngology, pediatric pulmonology, and tracheostomy team specialists, make it a great environment for this study. Our institution has recorded an average of 45 pediatric tracheostomies annually over the past five years. The average hospital stay for these patients is approximately 30 days, varying with the underlying pathology and postoperative course. Regarding emergencies, an average of 8–10 tracheostomy-related emergencies occur each year, as documented in cases of tube dislodgement, obstruction, and bleeding.

All 56 pediatric residents in different years of residency were invited to participate anonymously, and none were excluded. The study was conducted during the residents' designated half-academic education day to facilitate their attendance. The participation of pediatric residents in data collection for the study was on a voluntary basis, even though they were required to attend the session as part of their pediatric residency program. All participants gave consent to participate in the study and were told that the collected data would be used for research. They also had a right to exit the study without giving any reason.

The participants finished a self-assessment questionnaire to determine their confidence level and took an objective knowledge test on routine as well as emergency pediatric tracheostomy care. They also participated in a hands-on simulated routine change of the tracheostomy tube before and after a visit to a targeted tracheostomy workshop.

### 2.1 Self-assessment of confidence levels

The self-assessment questionnaire assessed the confidence level based on questions on training level, previous tracheostomy training, and statements about routine and emergency tracheostomy care using a 5-point Likert scale (1 = strongly disagree, 2 = disagree, 3 = neutral, 4 = agree, and 5 = strongly agree). The questions were designed to measure the levels of confidence of active participants and are provided in Supplementary Appendix S1.

### 2.2 Objective knowledge assessment

The objective test was designed after consultation with the tracheostomy care team (three certified nurses, a pediatric pulmonologist, and an otolaryngologist) in our hospital. There were 10 multiple-choice questions about the following subjects: general physiological changes related to tracheostomy, routine care, suctioning procedures, emergency care, acute and chronic issues, and tracheostomy indications. Currently, no instruments have been approved for measuring routine and emergency tracheostomy knowledge. Therefore, we adapted our objective knowledge assessment examination from previous adult medicine studies (12, 14) following the most recent clinical consensus statement, which is included in Supplementary Appendix S2 (16–19). The objective knowledge test and skills checklist were pre-tested and reviewed by a multidisciplinary panel of expert pediatric pulmonologists, otolaryngologists, and certified pediatric tracheostomy nurses. To ensure face and content validity, feedback was integrated through iterative rounds of review. We applied a modified Delphi approach, and experts from the relevant disciplines reviewed and developed questionnaire items to achieve consensus on their appropriateness and clarity. This ensured that the assessment instrument comprehensively addressed the important domains of tracheostomy care knowledge.

### 2.3 Hands-on routine tracheostomy change simulation assessment

All participants were required to demonstrate their clinical skills through the ability to conduct routine tracheostomy tube changes on a pediatric mannequin. Each participant was assessed before and after attending the tracheostomy training workshop. The hospital's tracheostomy team developed a checklist that included all the necessary steps to achieve the required competence in changing the pediatric tracheostomy tube. This checklist was constructed after a thorough literature review and is currently used in our hospital for training sessions. It is provided in Supplementary Appendix S3 (16–19).

The tracheostomy team reviewed all course materials for adequacy and accuracy. In addition, the self-assessment questionnaires administered before and after the workshop, the objective knowledge assessment, and the checklist for performing hands-on routine tracheostomy changes were all completed electronically using Google Forms.

### 2.4 Intervention

All participants attended a targeted tracheostomy care educational workshop carried out by the tracheostomy care team in our hospital during the pediatric half-academic day. The workshop included a theoretical session and practical simulation-based session. The theoretical review included a live Microsoft PowerPoint presentation that discussed pediatric airway anatomy, indications for tracheostomy, routine care procedure of tracheostomy, acute and chronic complications, as well as protocols for decannulation and emergency airway management.

The practical component was a hands-on simulation based training with pediatric mannequins and all the equipment and tools of routine tracheostomy change. This session started with an introduction of the required tools and equipment, followed by a demonstration of the normal way of changing a routine tracheostomy tube together with an appreciation of the emergency management of possible obstructions that could be encountered in the process of tracheostomy change. Subsequently, the participants were asked to practice their clinical skills on the mannequin with the tracheostomy care team observing.

### 2.5 Post-workshop evaluation

The end of the workshop required all participants to complete a workshop evaluation sheet, which contained four questions on a 5-point Likert scale. This form included questions about the usefulness of the workshop, its impact on their clinical practice, and whether they would recommend the workshop to other residents. There was a blank space for additional comments and suggestions for improving the content of future workshops. The form is provided in Supplementary Appendix S4.

### 2.6 Statistical analysis

Descriptive parameter statistics are shown as a value (percent) for categorical variables and as the mean and standard deviation (SD) for continuous variables. Mean scores on the hands-on simulated tracheostomy care test prior to and following the workshop and the mean differences between pre- and post-workshop self-assessment comfort level and objective knowledge test scores were compared using a paired samples *t*-test.

All the data were analyzed using SPSS (V 20; IBM, Chicago, Illinois, USA). All the statistical tests were two sided, and cases with an error probability < 5% were considered to be significant.

## 3 Results

Of the 56 pediatric residents invited to participate, 42 completed pre- and post-tracheostomy workshop questionnaires. The residents were fairly distributed among postgraduate years (PGY). Of the 42 participants, there were 12 (28.6%) PGY1 residents, 11 (26.2%) PGY2 residents, 8 (19.0%) PGY3 residents, and 11 (26.2%) PGY4 residents. The pediatric residents had limited experience with tracheostomy care; none had received formal training in tracheostomy care during their training, nor had they ever changed a tracheostomy tube on their own. Only eight residents (19.0%) had observed an elective tracheostomy tube change, and one resident had watched a video demonstration of a tracheostomy tube insertion.

Thirty-two (76.2%) participants agreed that pediatric residents are not adequately prepared to manage patients with a tracheostomy tube, while the rest felt neutral. Additionally, while 31 (73.8%) participants agreed that tracheostomy tube management should be included in the pediatric residency curriculum, 9 were neutral, and 2 disagreed.

### 3.1 Self-assessment of confidence levels

**Figure 1:**
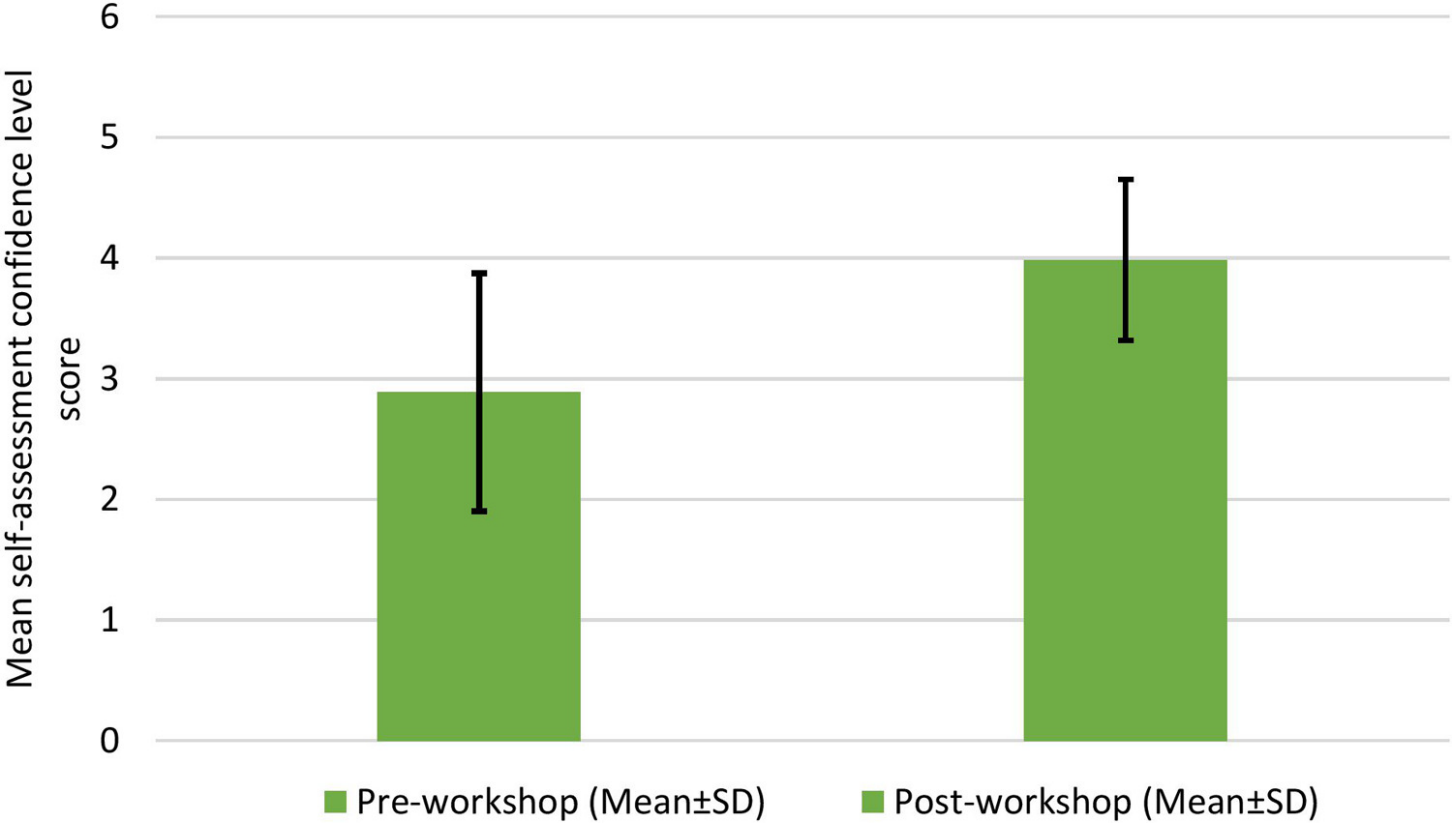
Overall results of the questionnaires administered before and after the workshop for the self-assessment of confidence levels.

**Figure 2:**
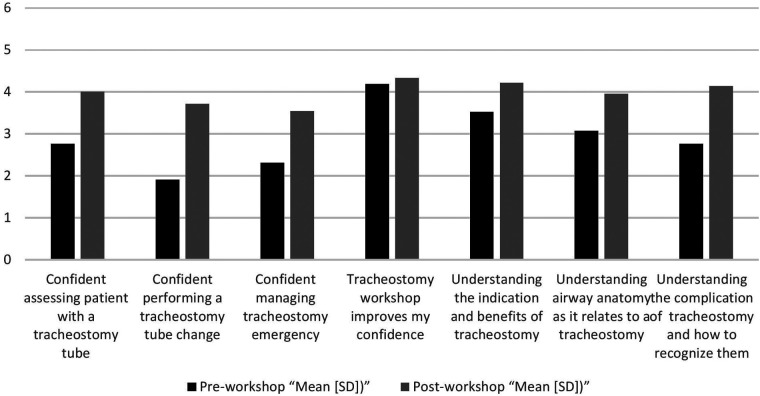
The responses to individual questions from the questionnaires administered before and after the workshop for the self-assessment of confidence levels.

Every resident answered a self-assessment questionnaire indicating their level of confidence before and after the workshop. Prior to the tracheostomy training workshop, pediatric residents demonstrated a mean (±SD) confidence level of 2.9 ± 9.9 on a 5-point Likert scale in the seven categories assessed on the self-assessment questionnaire. Following the completion of the tracheostomy training workshop, the mean (±SD) confidence level increased (3.9 ± 0.66; *p* < 0.001). The overall pre- and post-workshop scores of self-assessment of confidence levels are summarized in Figure 1. The responses to the individual questions of the pre- and post-workshop self-assessment questionnaire on confidence level are presented in Figure 2.

As shown in Figure 2, the pre- and post-workshop self-assessment questionnaire findings demonstrated an improvement in confidence levels in different areas following workshop attendance. Specifically, confidence in evaluating a patient with a tracheostomy tube increased from a mean rating of 2.8 before the workshop to 4.0 afterwards. Similarly, competence in tracheostomy tube change improvement was significant, increasing from 1.9 to 3.8. Participants' confidence in managing tracheostomy complications also increased from 2.2 to 3.3. Global confidence, as indicated by the statement “Tracheostomy workshop enhances my confidence” similarly improved by a minimal but encouraging amount from 4.1 to 4.2. In addition, awareness of the indications and benefits of tracheostomy increased from 3.3 to 4.1, while airway anatomy knowledge related to tracheostomy improved from 3.1 to 4.0. Finally, confidence in recognizing and valuing tracheostomy complications improved significantly from 2.9 to 4.1. Taken together, these results indicate the effectiveness of the workshop in increasing participants' confidence and proficiency in tracheostomy-related skills.

### 3.2 Objective knowledge assessment

**Figure 3:**
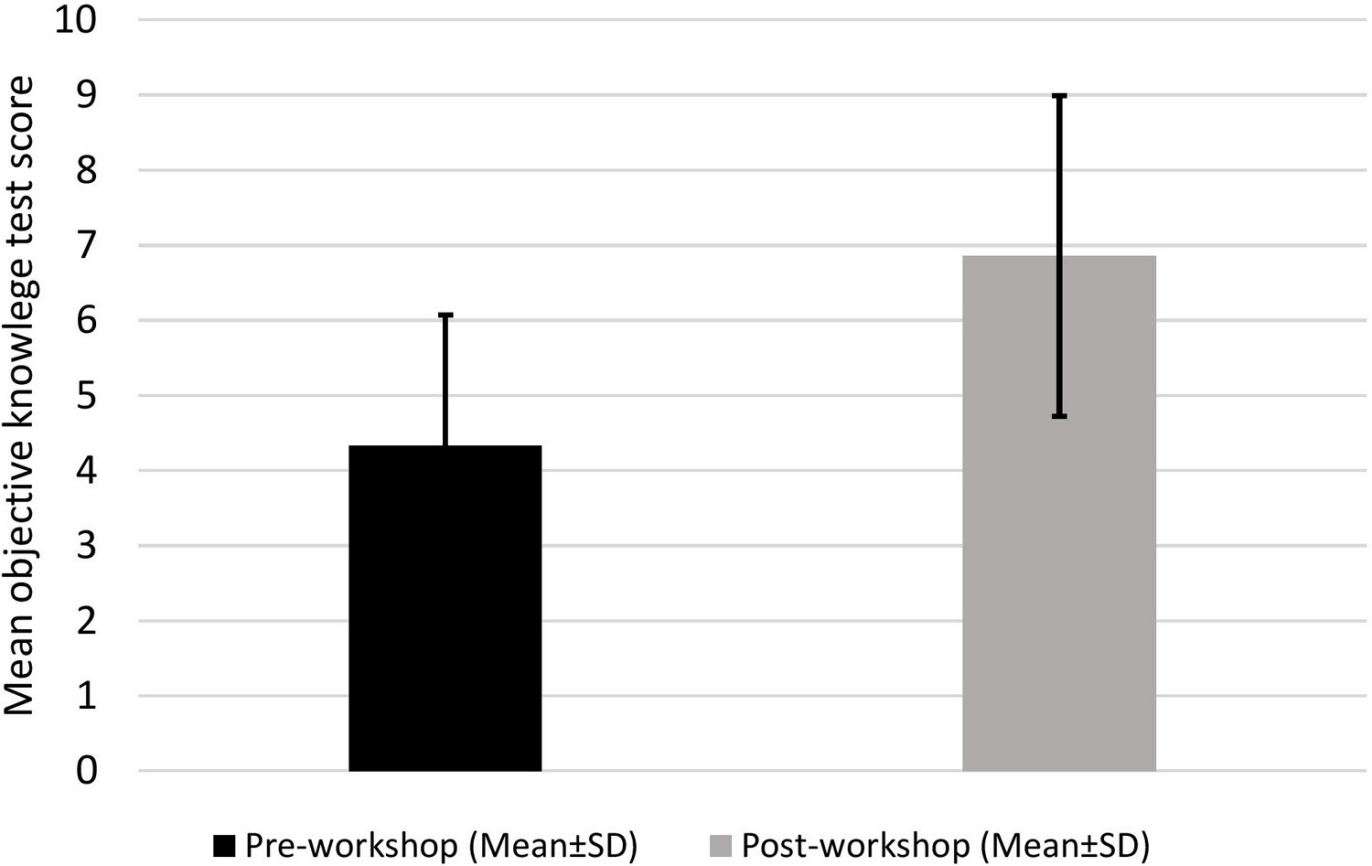
Overall results of the objective knowledge tests administered before and after the workshop.

All residents completed pre- and post-workshop objective knowledge tests consisting of 10 multiple-choice questions regarding the indications, advantages, potential complications, and routine and emergency management of a tracheostomy tube. Before attending the tracheostomy training workshop, pediatric residents reported a mean test score (±SD) of 4.33 (±1.79). The objective knowledge test mean score increased significantly to a mean score (±SD) of 6.86 ± 2.14 and *p* < 0.001 after residents attended the tracheostomy training workshop. The overall scores of the pre- and post-workshop objective knowledge test are summarized in Figure 3.

### 3.3 Hands-on routine tracheostomy change simulation assessment

**Figure 4:**
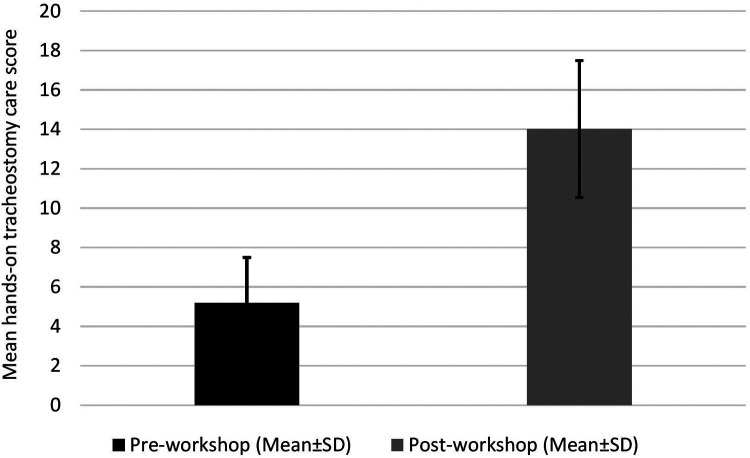
Overall scores of the hands-on tracheostomy care questionnaires administered before and after the workshop.

All residents performed hands-on tracheostomy care using pediatric mannequins before and after the workshop. Residents were assessed using a checklist of 18 steps required for completing a routine tracheostomy tube change by the tracheostomy care team in our hospital. Based on their performance of a baseline respiratory assessment, knowledge of different types of tracheostomy tubes and components, knowledge of procedures involved in checking equipment, ability to suction correctly, and knowledge of how to remove an existing tracheostomy tube and insert a new one, the participants' reaction to the final score was assessed. Before attending the tracheostomy training workshop, pediatric residents reported a mean tracheostomy care score (±SD) of 5.2 (±2.31). the hands-on skills demonstrated by the residents in the practice of tracheostomy care after the workshop improved substantially, with a post-workshop mean score (±SD) of 14.0 ± 3.48 (*p* < 0.001). Figure 4 summarizes the overall pre- and post-workshop scores.

### 3.4 Workshop evaluation

Approximately 13 residents (31.0%) strongly agreed and 21 (50.0%) agreed that the tracheostomy training workshop was an effective training tool to improve their knowledge and confidence in managing patients with a tracheostomy tube. Moreover, 9 residents (21.4%) strongly agreed and 26 (61.9%) agreed that the workshop would change their future practice in managing patients with a tracheostomy tube. Thirty-three residents (78.5%) considered recommending the tracheostomy training workshop to residents in other hospitals.

## 4 Discussion

Compared with adult patients, pediatric patients with tracheostomies are at significantly higher risk of airway obstruction and life-threatening complications such as tube blockage or accidental decannulation (4, 20, 21). Despite this increased risk, in our study, 76% of pediatric residents reported feeling unprepared to handle tracheostomy emergencies, highlighting a critical training gap. This aligns with earlier research that shows that nonsurgical healthcare professionals from various specialties tend to experience a lack of confidence and clinical ability needed to deliver effective tracheostomy care because of inadequate knowledge and exposure (12–15). These observations highlight the critical need for incorporating tracheostomy management as a central competency in pediatric residency training.

To address this gap, we conducted a workshop to reduce residents' anxiety related to tracheostomy care by providing theoretical knowledge and practical experience. Previous studies have shown significant gaps in understanding tracheostomy care among healthcare providers, including pediatric trainees (11–15). This type of research continually demonstrates that certain educational interventions can enhance knowledge, skills, and confidence for tracheostomy care.

After the workshop, we observed an appreciable improvement in residents' confidence, medical knowledge, and clinical practices of tracheostomy care. These results underscore the importance of providing tailored educational programs that address the specific needs of pediatric residents and improve their ability to assess patients with tracheostomy tubes and to manage associated emergencies. Interestingly, while confidence levels generally increased, the improvement related to the statement “The tracheostomy workshop would improve my confidence” did not achieve statistical significance. This may reflect that tracheostomy management was a novel experience for many residents, suggesting that building confidence requires ongoing exposure and practice.

Identifying the gap in our pediatric residency program and designing a targeted workshop were critical steps to increase participant engagement and create an interactive, hands-on learning environment. Residents appreciated the hands-on simulation of routine tracheostomy care in small groups, facilitating skill acquisition, knowledge reinforcement, and confidence building.

Participants viewed the workshop as a valuable educational experience that effectively met their needs. The majority expressed that the workshop would positively impact their approach to tracheostomy management and recommended it to colleagues in pediatric training who could not attend. As previously mentioned, reliance on healthcare technologies, including tracheostomies, is increasing in pediatric patients, and complications related to tracheostomy care may not be preventable. We assume that if residents increasingly familiarize themselves with the routine and emergency care of children undergoing tracheostomy, the health situation of such children will be more positive. It is expected that this improved competence will lead to a reduction in tracheostomy-related morbidity and mortality.

The current research validates the increasing demand for systematic simulation-based training to develop residents' skills and ensure better patient safety. The literature further emphasizes the dynamic nature of pediatric tracheostomy care and the need for innovative training models. Truitt et al. (22) highlighted knowledge gaps in family caregivers managing outpatient tracheostomy-related emergencies, further supporting the need for formal education among caregivers and healthcare professionals. Abbas et al. (23) introduced a virtual reality-based approach to remotely train providers in pediatric emergency tracheostomy skills, aiming to address geographical and logistical barriers to training. Additionally, Tawfik et al. (24) validated a mobile application designed to assess pediatric tracheostomy emergency simulations, supporting the utility of digital tools in skill assessment and reinforcement. Moreover, Schiff et al. (25) contrasted operant learning with the conventional demonstration teaching of tracheostomy tube changes and found that learner-centered instruction could improve skill acquisition. These results confirm and justify the applicability of the simulation-based workshop as a tool to enhance resident proficiency in the management of tracheostomy care and emergencies.

For all healthcare professionals who are working in pediatric care, it is necessary to improve the competence and skills related to tracheostomy management. These improvements should not be limited to pediatric residents. In addition, the literature shows a notable deficit in nurses' competencies in pediatric tracheostomy management, as evidenced by a recent study conducted in Saudi Arabia (9).

There are some limitations to this research. First, there is a problem with the absence of a validated questionnaire to measure confidence and knowledge in tracheostomy care management. To address this, we conducted a comprehensive review of existing literature on clinical practice guidelines and consulted with our expert tracheostomy team, including clinical nurses, an otolaryngologist, and a pediatric pulmonologist (15–19).

Second, our sample size was rather small, which may reduce its applicability. Nonetheless, this is the first attempt of its kind to discuss this issue within pediatric curricula in the Kingdom of Saudi Arabia. It may serve as a basis for future research to enhance physicians' competencies in this area.

Third, the morbidity of this study was applied to only pediatric residents of one institution. The knowledge, skills, and confidence that residents might have in tracheostomy care can be studied in future research projects with other residency programs where such patients are treated, such as internal medicine, anesthesia, critical care, and surgery. Researches have previously cited similar training gaps, and focused training programs have been shown to bridge said gaps (11, 13, 14).

Additionally, the data were collected within a single training center, potentially confining the outcomes' applicability to other institutions with different training conditions. Only 42 residents out of 56 completed both pre- and post-workshop questionnaires, introducing the risk of participation bias. Those who completed the questionnaire may have been more interested in or already familiar with the topic, which might have influenced the results.

Finally, more research is required owing to the lack of many long-term investigations of educational programs' impact on patient care outcomes. Future studies must explore how educational interventions influence patient outcomes and healthcare workers' skills in the long term.

Participants expressed positive feedback about the simulation-based tracheostomy workshop because it enhanced pediatric resident knowledge and skills; however, a permanent training program failed to establish itself. The simulation workshop was a singular educational session during the residents' academic time. The pediatric residency curriculum from the Saudi Commission for Health Specialties lacks tracheostomy care as an essential competency. The data highlight an urgent need to establish this training as a regular part of the residency program curriculum. Future studies should include this training program into the annual academic schedule as a formal component of resident education for airway management.

## 5 Conclusion

This research identified a high lack of confidence, skill, and competence among pediatric residents within our tertiary institution regarding tracheostomy care and the management of tracheostomy-related emergencies. Furthermore, we demonstrated that providing a carefully implemented and targeted tracheostomy training workshop tailored to the specific needs of pediatric residents could effectively enhance their confidence, knowledge, and skills. Based on these findings, we recommend incorporating pediatric tracheostomy care and management into the pediatric residency airway management curriculum.

## Data Availability

The raw data supporting the conclusions of this article will be made available by the authors, without undue reservation.
