# Supplementary material for: Assessing and enhancing pediatric residents’ knowledge and skills in tracheostomy care through simulation-based training
Source: Front Pediatr. 2025 Jul 15;13:1551517. doi: 10.3389/fped.2025.1551517 (PMC12303936; doi:10.3389/fped.2025.1551517)
Supplement: Supplementary file 1 [file Table1.docx]

Supplementary Material

# Appendix 1: Self-assessment of confidence levels

Candidate number:

Please answer the following questions as best as you can:

Residency Level:

PGY-1 PGY-2 PGY-3 PGY-4 Other:

Have you ever received tracheostomy care training?

Yes No

- I have observed a tracheostomy tube change (check all that apply)

o I have never observed a tracheostomy tube change

o On a mannequin

o Elective tracheostomy tube change

o Emergent tracheostomy tube change

o Other, please specify:

For the following questions, please answer to the best of your knowledge:

1- I feel confident in assessing a patient with a tracheostomy tube.

(1) Strongly disagree

(2) Disagree

(3) Undecided

(4) Agree

(5) Strongly agree

2- I feel confident in performing tracheostomy tube changes.

(1) Strongly disagree

(2) Disagree

(3) Undecided

(4) Agree

(5) Strongly agree

3- I feel confident in managing an airway emergency in a patient who has a tracheostomy.

(1) Strongly disagree

(2) Disagree

(3) Undecided

(4) Agree

(5) Strongly agree

4- Tracheostomy training that includes more hands-on teaching care would improve my confidence in emergency situations.

(1) Strongly disagree

(2) Disagree

(3) Undecided

(4) Agree

(5) Strongly agree

5- I understand the indications for and potential benefits of a tracheostomy.

(1) Strongly disagree

(2) Disagree

(3) Undecided

(4) Agree

(5) Strongly agree

6- I understand the airway anatomy as it relates to a tracheostomy, cricothyroidotomy, and a laryngectomy.

(1) Strongly disagree

(2) Disagree

(3) Undecided

(4) Agree

(5) Strongly agree

7- I understand the potential complications of a tracheostomy and how to recognize them.

(1) Strongly disagree

(2) Disagree

(3) Undecided

(4) Agree

(5) Strongly agree

8- My pediatric residency training adequately prepared me for an emergency tracheostomy tube change.

(1) Strongly disagree

(2) Disagree

(3) Undecided

(4) Agree

(5) Strongly agree

9- I think that tracheostomy tube care/change should be included as one of the objectives in the pediatric residency program curriculum.

(1) Strongly disagree

(2) Disagree

(3) Undecided

(4) Agree

(5) Strongly agree

**2 Appendix 2: Objective knowledge assessment test**

1. Which statement is true regarding the type of pediatric tracheostomy tube?
   1. Cuffless tubes are preferred for neonates and pediatric patients
   2. Cuffed tubes prevent gastric aspiration
   3. Cuffed tubes can be used in paralyzed children to minimize the risk of aspiration
   4. Cuffed tubes are associated with a lower risk of damage to tracheal wall mucosa
   5. Fenestrated tubes are associated with a lower risk of granuloma formation
2. Which statement is true when changing a tracheostomy tube?
   1. The first tracheostomy tube change should be performed by the ENT specialist one month after insertion
   2. Tracheostomy tube changes should be routinely performed every week
   3. During a tracheostomy tube change, the patient should lie in a supine position with a rolled towel under the neck and shoulders
   4. The tracheostomy tie should be very tight to hold the tracheostomy tube in place
   5. Normal saline should routinely be instilled into the tracheostomy tube before the tube change to liquefy secretions
3. Regarding the physiological changes following a tracheostomy insertion, the tracheostomy tube is going to:
   1. Increase the anatomical dead space of the respiratory system
   2. Decrease the humidification of inspired air
   3. Prevent patient's ability to speak
   4. Prevent patient's ability to eat
   5. Increase the temperature of inspired air
4. What should be avoided if the tracheostomy tube cannot be replaced with the same size during an attempt to change it?
   1. Force the same size tracheostomy tube back into the stoma
   2. Use a bag-valve mask device to gently ventilate the patient after covering the stoma
   3. Call the emergency response team
   4. Try to replace the tracheostomy tube with a smaller one
   5. Suction the stoma and administer oxygen
5. Which statement is true regarding tracheostomy care?
   1. A suctioning procedure should be performed regularly
   2. High suction pressure is recommended to remove mucus effectively from the tracheostomy tube
   3. The duration of suctioning should be less than 60 seconds to reduce the risk of hypoxia and trauma
   4. Bacteria can be introduced during tracheostomy suctioning and lead to infection and fistula formation
   5. Normal saline should be instilled routinely to liquefy secretions
6. All of the following signs suggest an obstructed tracheostomy tube except
   1. Grunting
   2. Cyanosis
   3. Diaphoresis
   4. Loud crying
   5. Stridor
7. Which one of the following is not an indication for performing a tracheostomy in a child?
   1. Child with upper airway obstruction (laryngeal hemangioma)
   2. Neonate with hypotonia who becomes ventilator dependent
   3. Child with central hypoventilation
   4. Teenager with bronchiectasis
   5. A child with consistent secretions that are difficult to suction and lead to O2 desaturation
8. Which of the following is the least important consideration when changing a tracheostomy tube?
   1. The size of the tube
   2. The presence of a cuff
   3. The length of the tube
   4. The presence of an obturator
   5. An underlying condition
9. Which of the following is not an advantage of tracheostomy?
   1. Allows for assisted ventilation
   2. Allows speaking using a special valve
   3. Reduces the risk of airway infections
   4. Facilitate the suctioning of excessive secretions
   5. Relieves an upper airway obstruction
10. The most important step before routine tracheostomy care is?
    1. Oxygenation
    2. Hand hygiene
    3. Preparing the suction
    4. Getting the ambo-bag ready
    5. Placing the patient in a supine position

**3 Appendix 3: Hands-on routine tracheostomy change simulation assessment (checklist):**

| Observation | Able to perform | Unable to perform | Comments |
| --- | --- | --- | --- |
| 1. Wash hands and review universal precautions |  |  |  |
| 2. Identify an assistant to help position, assist in tube change |  |  |  |
| 3. Perform baseline respiratory assessment |  |  |  |
| 4. Prepare new tracheostomy tube:  a. Check cuff for leaks (for cuffed tube)  b. Insert obturator  c. Lubricate the tube with water-soluble gel |  |  |  |
| 5. Position the patient by exposing the neck and straightening the airway;  place a rolled towel under the patient’s shoulders and neck |  |  |  |
| 6. Assess the patient for oxygenation and respiratory distress during the entire procedure |  |  |  |
| 7. Old tube removal  Cuffed tracheostomy: deflate cuff (as appropriate); changer holds old tracheostomy tube in place while ties are cut or undone; on the count of three, the assistant will remove the old tube with a gentle steady motion, outward and downward following the natural curvature of the tracheostomy tube |  |  |  |
|  |  |  |  |
| 8. The changer will insert the new tracheostomy tube; use a gentle, arcing motion following the natural curvature of the tube; do not force |  |  |  |
| 9. Remove the obturator immediately; provide oxygen, ventilation, or suction as needed while holding the tube in place |  |  |  |
| 10. Cuffed tracheostomy: inflate the cuff as appropriate; check the leak; the leak is heard with the stethoscope |  |  |  |
| 11. Auscultate bilateral breath sounds using the stethoscope; observe for correct placement of the tube, chest movement, color, vital signs, bilateral breath sounds |  |  |  |
| 13. Secure the tube with the tube holder |  |  |  |
| 14 Assistant holds new tube in place while changer attaches ties; leave one finger width between ties and neck |  |  |  |
| 15. Monitor respiratory status and response to procedure |  |  |  |

**4 Appendix 4: Post-workshop evaluation**

1- Do you think that tracheostomy tube care/change should be included as one of the objectives in the pediatric residency program curriculum?

- - 1. Strongly disagree
    2. Disagree
    3. Undecided
    4. Agree
    5. Strongly agree

2- This workshop was helpful to increase my knowledge and confidence regarding managing patients with tracheostomy.

1. Strongly disagree
2. Disagree
3. Undecided
4. Agree
5. Strongly agree

3 -This workshop changed my practice regarding managing patients with tracheostomy tubes.

1. Strongly disagree
2. Disagree
3. Undecided
4. Agree
5. Strongly agree

4- I would recommend this workshop to all pediatric residents in other programs (other than NGHA).

1. Strongly disagree
2. Disagree
3. Undecided
4. Agree
5. Strongly agree

5- Would you add any comment regarding your confidence in dealing with children who have a tracheostomy tube? (Free text)
